# Assessing the Effects of Thiazole-Carboxamide Derivatives on the Biophysical Properties of AMPA Receptor Complexes as a Potential Neuroprotective Agent

**DOI:** 10.3390/molecules29133232

**Published:** 2024-07-08

**Authors:** Mohammad Qneibi, Mohammed Hawash, Sosana Bdir, Mohammad Bdair, Samia Ammar Aldwaik

**Affiliations:** 1 Department of Biomedical Sciences, Faculty of Medicine and Health Sciences, An-Najah National University, Nablus P400, Palestine; s12027767@stu.najah.edu (S.B.); mohammad.bdair02@gmail.com (M.B.); samiaammardweik@gmail.com (S.A.A.); 2 Department of Pharmacy, Faculty of Medicine and Health Sciences, An-Najah National University, Nablus P400, Palestine; mohawash@najah.edu

**Keywords:** AMPAR, desensitization, deactivation, thiazole, carboxamide

## Abstract

An optimal balance between excitatory and inhibitory transmission in the central nervous system provides essential neurotransmission for good functioning of the neurons. In the neurology field, a disturbed balance can lead to neurological diseases like epilepsy, Alzheimer’s, and Autism. One of the critical agents mediating excitatory neurotransmission is α-amino-3-hydroxy-5-methylisoxazole-4-propionic acid receptors, which are concerned with synaptic plasticity, memory, and learning. An imbalance in neurotransmission finally results in excitotoxicity and neurological pathologies that should be corrected through specific compounds. Hence, the current study will prove to be an evaluation of new thiazole-carboxamide derivatives concerning AMPAR-modulating activity and extended medicinal potential. In the current project, five previously synthesized thiazole-carboxamide derivatives, i.e., TC-1 to TC-5, were used to interact with the AMPARs expressed in HEK293T cells, which overexpress different subunits of the AMPAR. Patch-clamp analysis was carried out while the effect of the drugs on AMPAR-mediated currents was followed with a particular emphasis on the kinetics of inhibition, desensitization, and deactivation. All tested TC compounds, at all subunits, showed potent inhibition of AMPAR-mediated currents, with TC-2 being the most powerful for all subunits. These compounds shifted the receptor kinetics efficiently, mainly enhancing the deactivation rates, and hence acted as a surrogate for their neuroprotective potentials. Additionally, recently published structure–activity relationship studies identified particular substituent groups as necessary for improving the pharmacologic profiles of these compounds. In this regard, thiazole-carboxamide derivatives, particularly those classified as TC-2, have become essential negative allosteric modulators of AMPAR function and potential therapeutics in neurological disturbances underlain by the dysregulation of excitatory neurotransmission. Given their therapeutic effectiveness and safety profiles, these in vivo studies need to be further validated, although computational modeling can be further developed for drug design and selectivity. This will open possibilities for new drug-like AMPAR negative allosteric modulators with applications at the clinical level toward neurology.

## 1. Introduction

The excitatory and inhibitory neurotransmission equilibrium within the central nervous system (CNS) is fundamental to maintaining proper neuronal function in healthy individuals [1]. The imbalance of excitatory and inhibitory signals, whether from an increase in one or the decrease of another, results in the onset of various neurological diseases and the impairment of cognitive, motor, and behavioral functions [2,3,4,5]. α-amino-3-hydroxy-5-methylisoxazole-4-propionic acid receptors (AMPARs), the most abundant excitatory glutamate receptors in the brain, play a pivotal role in the processes of synaptic plasticity, memory formation, and learning [6,7,8,9]. The dysregulation of these receptors and excessive activity have been implicated in various debilitating neurological diseases such as epilepsy, neurodegenerative disorders such as Alzheimer’s, and developmental disorders such as Autism [10,11,12]. Therefore, the modulation of AMPAR activity through different compounds is important in creating therapeutic drugs to alleviate the stress on these individuals’ physical, mental, and financial well-being [13,14,15]. That is why understanding the intricate architecture of AMPARs is crucial for developing those NAMs.

AMPARs are ion-gated channels of four subunits (GluA1–GluA4) arranged in a tetrameric structure. The subunits, which come together as dimers of dimers, exhibit structural variability due to their potential combinations. The subunit consists of a C-terminal domain, an N-terminal domain, and a ligand-binding domain comprising two lobes, four loops, three transmembranes, and one reentrant [16]. Glutamate activates AMPARs, causing their ion channels to open quickly. This allows sodium ions to enter, rapidly depolarizing the postsynaptic membrane and transmitting excitatory impulses [17]. Most AMPAR formations allow calcium influx when activated, which causes N-methyl-D-aspartate receptors (NMDARs) to repel the magnesium, blocking their surface and causing the potentiation and surface trafficking of AMPARs. The trafficking of additional AMPARs subsequently causes excitotoxicity [18]. Excitotoxicity refers to cell death caused by intense glutamate exposure [19]. Excitotoxicity is not necessarily caused by the overexpression of excitatory receptors like AMPARs but can also result from a decrease in inhibitory receptors. In both cases, decreasing the excitatory receptors’ activity would decrease excitotoxicity and restore the balance of signals.

Structure–activity relationships (SARs) refer to the relationship between a molecule’s chemical or 3D structure and its biological activity. Understanding SARs allows researchers to optimize the pharmacological properties of compounds, making them more effective and selective in their action. The SARs between benzodiazepines and AMPARs have been extensively studied, leading to the development of potent therapeutic compounds like Perampanel [20,21,22]. The discovery of this compound did not deter the SAR investigations from being conducted; rather, it promoted them and provided a valuable foundation for researchers to create even stronger compounds that could be used to treat other neurological diseases [9,23,24,25].

**Figure 1:**
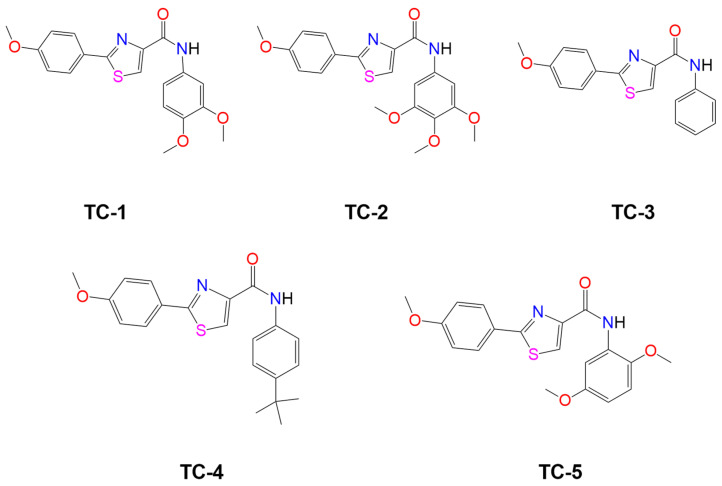
Chemical structures of thiazole-carboxamide derivatives.

Thiazole derivatives have emerged as prominent candidates for AMPAR modulation among the NAM families being explored. Thiazole rings, five-membered rings that contain nitrogen and sulfur, have been previously found to have an inhibitory effect on AMPAR biophysical gating properties and activity. In this study, we used previously synthesized five thiazole-carboxamide derivatives (TC-1 to TC-5) in our laboratory [26], starting from 2-(4-methoxyphenyl)thiazole-4-carboxylic acid, which was also synthesized by our team (Figure 1). Thiazole is also used in Riluzole, an ALS medication with neuroprotective properties approved by the FDA [27]. These compounds are expected to exhibit similar effects to Dr. Hawash’s previously studied carboxamide derivatives [28]. These previously synthesized compounds [26] exhibited strong NAM effects by reducing the current amplitude and desensitization rate while promoting the deactivation rate. By administering these compounds to GluA1, GluA1/2, GluA2, and GluA3 AMPARs, we hope to obtain further insight into their mechanism and aid in the pursuit of discovering effective therapeutic compounds for a multitude of neurodegenerative diseases.

## 2. Results

### 2.1. TC Compounds Potently Inhibit AMPA Receptor Subunits in Transfected HEK293T Cells

**Figure 2:**
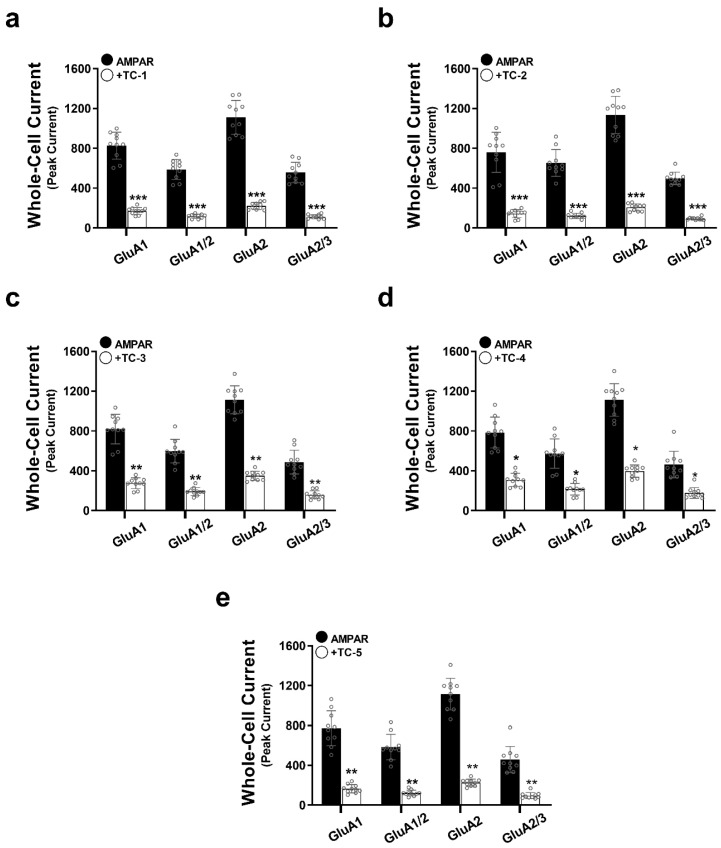
Inhibition of homomeric and heteromeric AMPA receptor subunits by TC compounds. The graphs illustrate the inhibition of homomeric and heteromeric AMPAR subunits by TC compounds. Whole-cell recordings of amplitude (**a**–**e**) were obtained from HEK293T cells expressing AMPAR subunits after treatment with 10 mM glutamate alone (black) and with glutamate plus TC compounds (white). The recordings were conducted at a membrane potential of −60 mV, pH 7.4, and a temperature of 22 °C. Statistical significance is denoted as follows: * *p* < 0.05; ** *p* < 0.01; *** *p* < 0.001.

The effect of five TC compounds on whole-cell currents of four AMPAR subunits (GluA1, GluA1/2, GluA2, and GluA2/3) was investigated using transfected HEK293T cells. Each compound was applied separately to each subunit, with experiments conducted on 8–10 cells per condition to ensure reproducibility. As depicted in Figure 2 and Supplementary Tables S1–S5, all five compounds significantly inhibited the tested subunits. At a glutamate concentration of 10 mM and a 500 ms protocol, approximately 95% of AMPAR channels were open. The A/A_I_ ratio was calculated to compare the results, where A represents normalized current readings and AI indicates the inhibited current following TC compound exposure. TC-2 demonstrated the most potent effect on all tested subunits, resulting in approximately a 5.5-fold decrease in AMPAR subunit activity. Following closely, TC-1 inhibited the tested subunits by 5-fold. TC-5, TC-3, and TC-4 exhibited inhibition of the whole-cell current by approximately 5-fold, 3-fold, and 2.5-fold, respectively.

**Figure 3:**
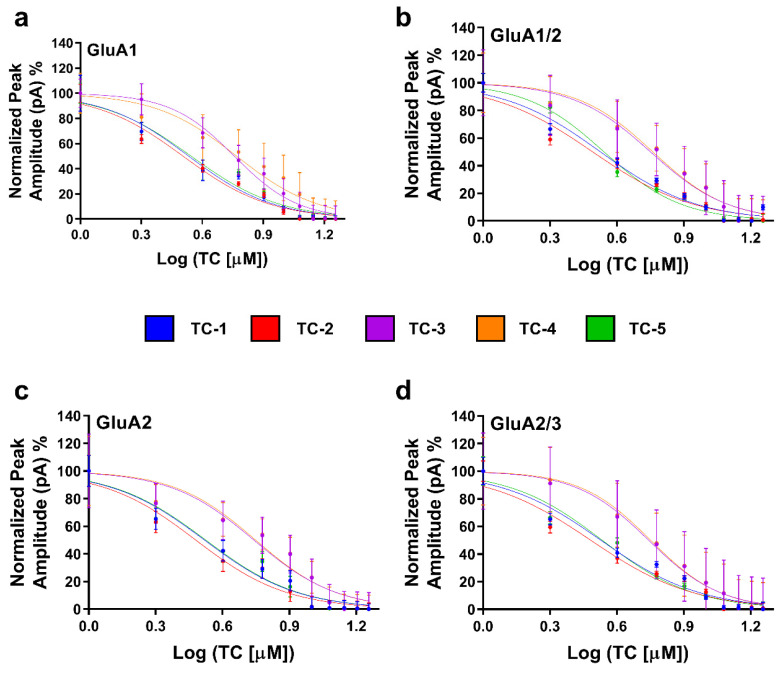
Dose–response curve of TC compounds on AMPA receptor subunits. The figures (**a**–**d**) illustrate the dose–response relationship between TC compounds and AMPAR subunits, represented as a curve against the logarithm of compound concentration. The experiments were conducted on HEK293T cells held at a membrane potential of −60 mV, maintained at pH 7.4, and a temperature of 22 °C. The error bars, which depict the standard error of the means, provide valuable information on the dispersion of the data.

The IC_50_ values were examined using GraphPad Prism software 9.5.0.730 (x64) (Table S6), which confirmed these findings. The IC_50_ values of TC substances were calculated by standardizing the peak current amplitudes using data obtained from 8–10 cells. Following a 500 ms exposure to the compounds, the cells were rinsed with glutamate for 20 s. Next, a solution containing just glutamate at a concentration of 10 mM was administered, and the resulting immediate induced electrical current was measured and documented (Figure 3). Results revealed that TC-2 pronounced inhibitory impact on GluA2, GluA2/3, GluA1/2, and GluA1 at low micromolar concentrations (3.02 µM, 3.04 µM, 3.1 µM, and 3.20 µM, respectively). Particularly noteworthy was TC-2′s superior efficacy in suppressing AMPAR currents. For instance, the IC_50_ for GluA2 was estimated at 3.3 µM when treated with TC-1 and 3.35 µM with TC-5, as depicted in the dose–response curve (Figure 3). Detailed IC_50_ values for all TC compounds are provided in Table S6.

### 2.2. Modulation of AMPA Receptor Kinetics by TC Compounds

**Figure 4:**
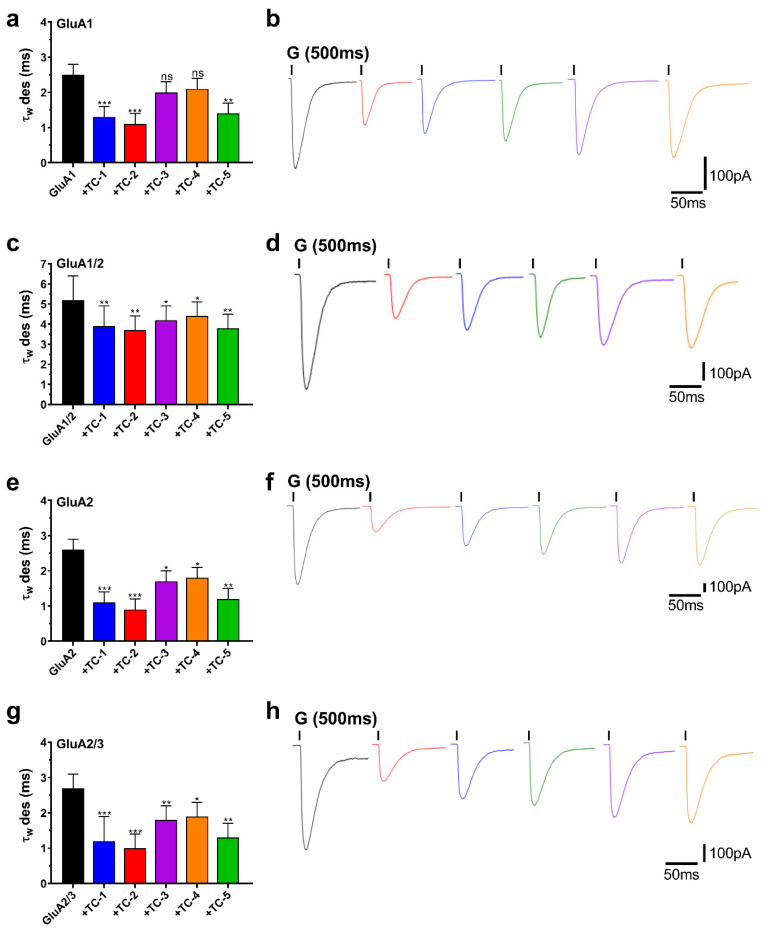
TC compounds modulate AMPA receptor desensitization rates. The graphical breakdown highlights their effects on specific subunits: (**a**,**b**) depict the impact on GluA1 desensitization, (**c**,**d**) focus on GluA1/2, (**e**,**f**) demonstrate interactions with GluA2, and (**g**,**h**) detail the modulation of GluA2/3. Notably, the distinct traces in (**b**,**d**,**f**,**h**) reveal significant alterations induced by the TC-1–5 series on AMPAR desensitization patterns. Throughout the experiments, whole-cell currents were recorded at −60 mV under pH 7.4 conditions at 22 °C. (*) indicates *p* value < 0.05, (**) for *p* < 0.01, and (***) for *p* < 0.001, while “ns” denotes non-significant results.

A thorough investigation was performed to assess the neuroprotective potential of the TC compounds. This analysis focused on the desensitization rates (τ_w_ des) of all tested subunits and compounds, as shown in Figure 4a,c,e,g. The influence of the five TC compounds on the desensitization traces is seen in Figure 4, namely in panels b, d, f, and h. TC-2 caused a substantial reduction in τ_w_ des in all receptor subunits, with decreases ranging from 2 to 3 times. GluA2 had the most prominent impact, while GluA1/2 exhibited the least robust reaction. TC-1 exhibited a comparable reduction in τ_w_ des across all examined subunits, with a roughly twofold drop found. However, the impact was significantly less pronounced for GluA1/2, with a decrease of around 1.3-fold.

In contrast, TC-5 caused a roughly twofold reduction in ιw des throughout the subunits. Meanwhile, TC-3 and TC-4 had little effect on τ_w_ des, while the influence on the GluA1 subunit was considered negligible. These results provide vital information on how each TC chemical affects desensitization rates differently across distinct receptor subunits.

**Figure 5:**
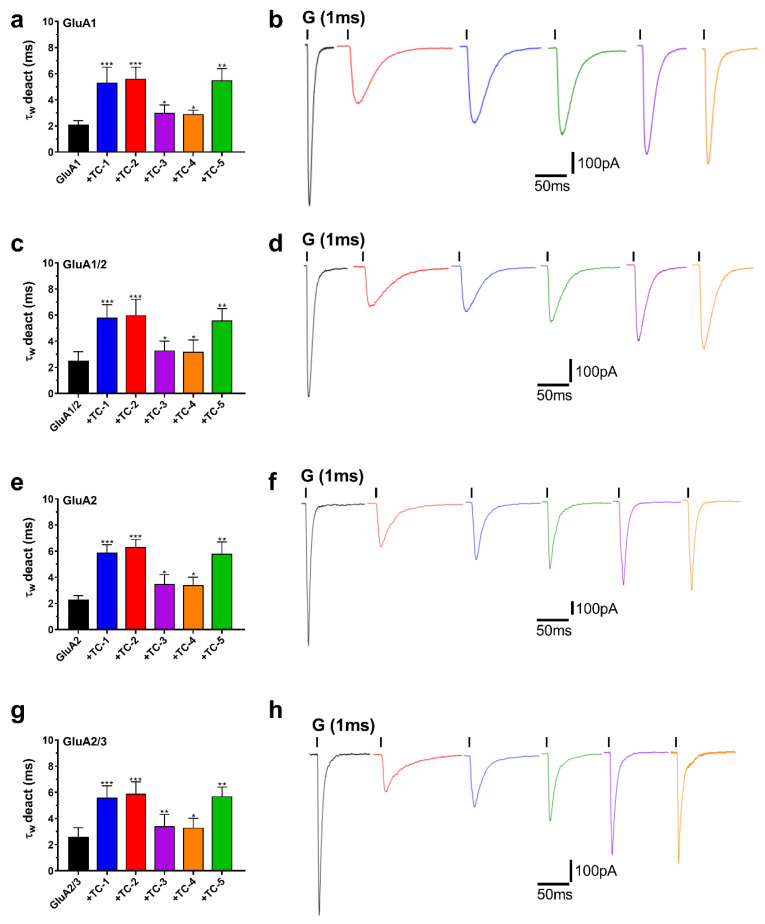
TC compounds modulate AMPA receptor deactivation rates. The graphical breakdown highlights their effects on specific subunits: (**a**,**b**) depict the impact on GluA1 desensitization, (**c**,**d**) focus on GluA1/2, (**e**,**f**) demonstrate interactions with GluA2, and (**g**,**h**) detail the modulation of GluA2/3. Notably, the distinct traces in (**b**,**d**,**f**,**h**) reveal significant alterations induced by the TC-1–5 series on AMPAR deactivation patterns. Throughout the experiments, whole-cell currents were recorded at −60 mV under pH 7.4 conditions at 22 °C. (*) indicates *p*< 0.05, (**) for *p*< 0.01, and (***) for *p*< 0.001, while “ns” denotes non-significant results.

In addition, an evaluation was conducted on the deactivation rates (τ_w_ deact) of all the examined chemicals to investigate their impact on the kinetics of deactivation. The chemicals were assessed for their effect on the deactivation kinetics of AMPAR subunits by briefly administering 10 mM glutamate for 1 ms to begin currents. This experimental setup allowed for the observation of τ_w_ deact under controlled conditions. As depicted in Figure 5 (panels b, d, f, and h), the results demonstrate the subsequent increase in τw deact in response to exposure to TC compounds. TC-2 notably increased τ_w_ deact for all tested subunits by nearly 2.5-fold, demonstrating a substantial effect. TC-1 exhibited a similar effect, albeit with a slightly lower increase in τ_w_ deact of around 2-fold. TC-5 induced an approximately 2-fold increase in τ_w_ deact across all four tested subunits. Conversely, TC-3 and TC-4 displayed weaker effects on increasing the deactivation rate, albeit still noteworthy (Figure 5a,c,e,g). These findings highlight the differential impact of TC compounds on deactivation kinetics across AMPAR subunits, shedding light on their potential neuroprotective mechanisms.

## 3. Discussion

The TC series, which combines the thiazole-4-carboxamide core, is one of a kind for AMPAR modulation. This scaffold, including methoxyphenyl substituents at various sites, is paramount for interaction with AMPARs. Several compounds structurally related to Thiazole-carboxamide derivatives, like Thiazolidinediones and Benzothiazoles, have been studied by different groups [29]. They have been studied for therapeutic use in diabetes, cancer, and neurodegenerative diseases. However, multiple side effects are reported. For example, weight gain and edema, as well as an increased risk of heart failure, have affected the clinical use and regulatory status of Thiazolidinediones. In preclinical and clinical studies, hepatocheatotoxicity was found in some Benzothiazoles. The problems are connected with these compounds’ off-target actions and metabolic byproducts. For example, thiazolidinediones activate peroxisome proliferator-activated receptor gamma (PPARγ), which can cause fluid retention and lipid metabolic changes. Benzothiazoles, on the other hand, have been reported to generate free radicals, which may lead to liver and heart toxicity [30,31].

A standout example is the creation of Rosiglitazone, a Thiazolidinedione used to help manage type 2 diabetes. Its effect on blood glucose is well established, but a link to an increased risk of myocardial infarction and cardiovascular death soon led to it being heavily regulated. All red flags resulted in thorough post-marketing research and governmental review to reevaluate the safety of this drug, which demonstrates the demanding balance between therapeutic advantages with side effects [32,33]. Lessons learned with this class of compounds ought to be considered when developing Thiazole-carboxamide derivatives as potential therapeutic agents. This early research stage is intended to uncover potential toxicological problems, especially off-target effects or metabolic byproducts. Phase III clinical trials should include comprehensive monitoring for adverse events to protect patients.

Moreover, learning the mechanisms responsible for these side effects can help design less hazardous mitigators. The methoxy groups, particularly, might be claimed to possess a dominant quality of enhancing the affinity of the compounds for their receptors. It was more likely because their electron-donating nature strengthened the interaction at the receptors’ binding sites. Because the methoxy groups of TC compounds are located with an appropriate distance and number around the molecule, these groups produce important molecular changes in conformation and electronic distribution. Hence, the more confined interaction with receptor binding sites of these substitutions is expected to induce changes in the efficacy and selectivity of the compounds towards different AMPAR subtypes leading to a finely tuned pharmacological profile for AMPAR modulation.

Modulating excitatory impulses is one of many approaches used to restore impaired balance in individuals with neurological diseases involving overexcitation, such as epilepsy or amyotrophic lateral sclerosis (ALS). Epilepsy or ALS could be a result of the overexcitation of excitatory receptors such as AMPARs. In order to inhibit or downregulate the effect of these excitatory signals, NAMs must be used. NAMs bind to a site different than glutamate on AMPARs, which triggers structural changes in the receptor, making it less responsive to glutamate [9,34]. Future studies analyzing the SARs of various NAMs with AMPARs will help develop potent and selective modulators that target AMPARs and decrease their response to glutamate [35]. The decreased response to glutamate is often exhibited in experiments as a decrease in amplitudes recorded, which reduces the excitatory signal and likelihood of a seizure to occur [36]. NAMs reduce the receptors’ activity and affect two important gating properties of AMPARs: desensitization and deactivation. Desensitization refers to the decreased response of a receptor to glutamate, meaning higher glutamate concentrations must occur for a response to be activated. Deactivation refers to when the receptor returns to its resting state after activation. If the rate of these processes is altered, they can affect excitotoxicity and thus help control seizures [9,28,37]. The targeted approach of using NAMs in AMPAR modulation is effective in treating epilepsy and potentially in other neurological diseases such as strokes, Huntington’s, and Alzheimer’s.

In the series, TC-2 is the relatively potent inhibitory compound for the AMPAR subunits owing to a structure that consists of a thiazole-4-carboxamide core connected to a 4-methoxyphenyl group and 3,4,5-trimethoxyphenyl group. This added pair of methoxy groups, with respect to the related compounds TC-1 and TC-3, provides a much larger increase in binding efficiency for receptor interaction, thus enabling a more prolonged and enhanced AMPAR engagement [38]. The increased hydrophobic interactions and enhanced electron density in the region around the phenyl rings can accommodate the binding enhancement [38]. Supported by the results of concurrent experiments with compounds such as the chlorophenyl 2,3-benzodiazepine analog series, these structural add-ons help provide the means for potent inhibition capabilities while also marking the clear path for further developments in drug design through site-targeted molecular modifications [21,22]. As guided by the studies, the molecular modifications are based on the principles of the basic structure–activity relationship, or SAR, that directs the modification. The importance of the substituent arrangement and electron characteristics for achieving an enhanced pharmacological action is the most important in the SAR principles.

Furthermore, the optimized structural design of TC-2 strategically fine-tunes binding affinity and selectively modulates key receptor kinetics, such as desensitization and deactivation rates, to influence AMPAR dynamics in a manner that permits nuanced control, potentially enhancing therapeutic efficacy. Insights from related studies on thiazole derivatives underscore the potential of these compounds in neuropharmacology, highlighting their diverse interactions with neural receptors and therapeutic possibilities [39,40,41].

The effects of TC compounds on both the currents and kinetics of AMPAR serve to highlight the above-stated complexities of the goal of targeting these receptors. While the reduction in current amplitude could serve as a potential mechanism in that less excitatory transmission should occur, the potential of increased activity from the prolonged deactivation serves to imply that the engagement of these receptors should be accomplished by a discriminating means of the use of AMPAR NAMs. This is imperative for further research in order to clarify the optimal means of engaging these receptors.

Building upon the knowledge gained from the present understanding of TC compounds and the manner in which they modulate AMPARs, certain directions of future research can be identified as being critical toward furthering our understanding and improving therapy, not just in epilepsy but in many other neurological diseases. Further detailed study into the specific effects of TC compounds on individual AMPAR subtypes may reveal subtype-dependent modulation mechanisms that may provide more selective therapeutic possibilities. In addition, performing in vivo studies into the efficacy and safety of such compounds in animal disease models can provide crucial information regarding their clinical applicability, including potential side effects and optimal dosing schedules. Investigation into the cooperative effects of TC compounds with currently available drugs and therapies for neurological diseases can also throw light on combination therapies that may be planned for synergistic advantages toward therapeutic outcomes while blocking the possibility of side effects. Further study into the long-term effects of AMPAR modulation on synaptic plasticity and network activity can provide clues into the evolution of therapeutic strategies that may be able to control the symptoms, co-morbidities, and overall neurological health in afflicted patients. Finally, future advanced computational modeling and molecular docking studies can provide clues into the dynamics of interaction of TC compounds with AMPARs that can be used to guide the rational design of next-generation NAMs with improved specificity and potency. With such directed pathways of research, we can hope to harness the amazing pharmacological talents of TC compounds for useful advances in the treatment of neurological diseases, with subsequent improvements in therapeutic efficacy in patients.

## 4. Materials and Methods

### 4.1. Chemistry

**Scheme 1:**
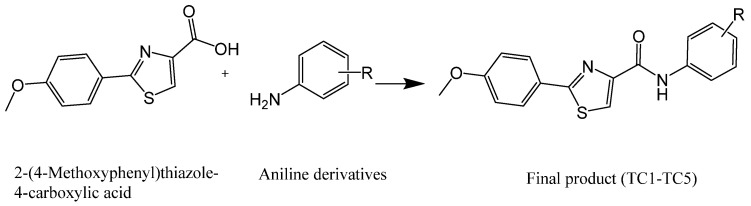
2-(4-Methoxyphenyl)thiazole-4-carboxylic acid+ appropriate aniline derivatives stirred in 20 mL DCM, then DMAP and EDC were added under inert gas and stirred for 48 h (R=H, 3,4-dimethoxy, 2,5-dimethoxy, 3,4,5-trimethoxy, and 4-tret-butyl).

The Thiazole carboxamide series TC-1-TC-5 was previously synthesized as outlined in Scheme 1. The coupling reaction to form these derivatives was afforded by using EDCI and DMAP as activating agents and covalent nucleophilic catalysts, respectively [26].

For detailed synthetic procedures of each compound, please refer to our previous study, which describes the synthesis of these compounds in detail [26].

### 4.2. Electrophysiological Insights

#### 4.2.1. Preparation of Plasmid DNA

The QIAGEN Plasmid Mini Kit was used to prepare up to 20 μg of high-copy plasmid DNA. A selective plate was streaked followed by the selection of a single colony. For a starter culture, the medium that was used was LB, which was inoculated containing the appropriate selective antibiotic. Afterward, the culture was incubated for approximately 8 h at 37 °C, which was later diluted with 3 mL selective LB medium. The culture was then placed in the incubator at 37 °C for roughly 12–16 h. Centrifugation was conducted followed by the resuspension of the formed pellet in order to harvest the bacterial cells. After the addition of 0.3 mL of Buffer P2, the sealed tube was inverted 4–6 times for homogeneity. Later, the tubes were centrifuged to obtain the supernatant containing the plasmid DNA. An amount of 1 mL Buffer QBT was used to equilibrate a QIAGEN-tip 20, the column could empty by gravity flow, then the supernatant was applied to the QIAGEN-tip 20, and by gravity flow, it entered the resin. Buffer QC was used to wash the QIAGEN-tip. This was followed with the elution of the DNA with 0.8 mL buffer QF, and then for precipitation purposes, isopropanol was also added. It was mixed and centrifuged immediately to carefully decant the supernatant. Ethanol was used to wash the DNA pellet, then centrifuged again, and the supernatant was carefully removed so as not to disturb the pellet. Finally, the pellet was air-dried, and the DNA was re-dissolved in a suitable volume of buffer. Running spectrophotometry at 260 nm, the quantitative analysis on agarose gel was used to calculate DNA concentration to determine the yield. A260 readings should lie between the values of 0.1 and 1.0 to judge the reliability of spectrophotometric DNA quantification, as outlined in our previous study [42].

#### 4.2.2. Expression and Recording in HEK293T Cells

The flip isoform was uniformly employed across all AMPAR subunits in this study. These subunits were subcloned into the pRK vector and expressed in Human Embryonic kidney cells sourced (Sigma, München, Germany). HEK293T cells were grown in Dulbecco Modified Eagle Medium (DMEM) (Sigma, München, Germany) containing 10% FBS (fetal bovine serum), 0.1 mg/mL streptomycin, and 1 mM sodium pyruvate (Biological Industries; Beit-Haemek, Israel). HEK293T cells were incubated at 37 °C and 5% CO_2_ was supplemented to the medium. It was subcultured twice a week until cells reached pass #20. The transfection reagent used was either jetPRIME (Polyplus: New York, NY, USA) or Lipofectamine 2000 (Invitrogen; San Diego, CA, USA). Cells were kept for 36 h. after transfection in 12-well plates. Then, they were replated on coverslips coated with Laminin (1 mg/mL; Sigma, Germany) for electrophysiology recordings. Cells were then seeded in Petri dishes in DMEM supplemented with 10% fetal calf serum and antibiotics and maintained in a humidified incubator at 37 °C and 5% CO_2_. Highly fluorescent cells were identified and selected for recording post-transfection.

HEK293T cells were recorded for 36–48 h, at a temperature of 22 °C, with a membrane potential of −60 mV. HEK293T cells were recorded 36–48 h after transfection using IPA (Integrated Patch Amplifier) via the catch clamp technique, at a temperature of 22 °C, with a membrane potential of −60 mV. SutterPatch Software v. 1.1.1 (Sutter Instruments, Novato, CA, USA) was used to digitize membrane currents for a short period. The sampling frequency was set to 10 kHz, and the low-pass filter was set to 2 kHz. Borosilicate glass was used to fabricate the patch electrodes with a resistance of 2–4 MΩ. The extracellular solution contained the following (values are in mM): 150 NaCl, 2.8 KCl, 0.5 MgCl_2_, 2 CaCl_2_, and 10 HEPES, adjusted to pH 7.4 with NaOH. The pipette solution contained the following (values are in mM): 110 CsF, 30 CsCl, 4 NaCl, 0.5 CaCl_2_, 10 Trypsin EDTA solution B (0.25%), EDTA (0.05%), and 10 HEPES, adjusted to pH 7.2 with CsOH. Using double barrel glass (theta tube), glutamate and the solutions of choice were rapidly administered, and the theta tube was mounted on a high-speed piezo solution switcher (Automate Scientific, Berkeley, CA, USA). After expelling the patch from the electrode to estimate the speed of solution exchange, the open tip potentials were recorded during the application of solutions of different ionic strengths. The 10–90% solution exchange was typically at 500 ms. The second barrel supplied the cells with glutamate and the derivatives individually after obtaining the current of each application. The solution exchange in the different tubes was interchangeable on a single cell to calculate the inhibition.

#### 4.2.3. Inhibition Assessment and Compound Administration

Inhibitory effects were quantified by comparing currents recorded following the administration of glutamate alone to those recorded when glutamate and the TC derivative were concurrently administered to the same cell. Preliminary experiments established the optimal concentration of TC derivatives (12 µM) to avoid cellular damage. The inhibitory action of TC derivatives on AMPAR subunits was found to plateau at 12 µM. The concentration was systematically increased in increments of 2 µM to limit cellular damage. After the application of TC derivatives, the cells underwent a 20 s wash. Following each TC application, a 10 mM glutamate solution was injected to assess the viability of the cells. Data analysis was performed using Igor Pro7 (Wave Metrics, Inc., Portland, OR, USA). Desensitization and deactivation rates were determined by fitting an exponential decay to the current from a 95% peak to baseline, with the weighted tau (τ_w_) calculated as τ_w_ = (τf × af) + (τs × as), where af and as represent the amplitudes of the fast (τf) and slow (τs) exponential components, respectively. This study’s detailed experimental dataset is presented in the references.

#### 4.2.4. Statistical Analysis

Data are presented as the mean ± standard deviation of the mean (SD), with the sample size (n = 8–10) reflecting the total number of tested HEK293T cells. Statistical distinctions among different groups and the control were assessed using a One-Way Analysis of Variance (ANOVA). Statistical significance was set at *p* < 0.05, with significance levels denoted as follows: (*) *p* < 0.05, (**) *p* < 0.01, (***) *p* < 0.001, and ns, not significant. The concentration–response relationships were modeled as composite curves adhering to the Hill equation via GraphPad Prism version 6.01 (GraphPad Software, San Diego, CA, USA). GraphPad Prism calculates IC_50_ values by fitting the data to a non-linear regression curve using the log(TC) vs. response-variable slope equation, which allows for determining the inhibitor concentration that gives the half-maximal response. All reported results are representative of a minimum of three independent experiments.

## 5. Conclusions

In conclusion, our investigation into thiazole-carboxamide derivatives, particularly the TC series, highlights their significant potential in modulating AMPARs and introducing innovative treatment approaches for neurological disorders. The unique structural characteristics of these compounds enhance receptor interactions, promising improved efficacy in conditions such as epilepsy and other neurodegenerative diseases. Insights gained from SAR studies have been pivotal in developing potent and selective AMPAR NAMs. As we progress, prioritizing in vivo toxicological evaluations and advanced computational modeling will be essential to deepen our understanding and optimize the drug development process. This research expands our knowledge of neuropharmacology and lays the groundwork for transformative clinical applications aimed at significantly improving the management and treatment of neurological disorders.

## Data Availability

The datasets generated during and/or analyzed during the current study are available on request from the corresponding author.

## Supplementary Materials

The following supporting information can be downloaded at: https://www.mdpi.com/article/10.3390/molecules29133232/s1.

## References

[1] Sohal V.S., Rubenstein J.L.R. (2019). Excitation-inhibition balance as a framework for investigating mechanisms in neuropsychiatric disorders. Mol. Psychiatry.

[2] Uzunova G., Pallanti S., Hollander E. (2016). Excitatory/inhibitory imbalance in autism spectrum disorders: Implications for interventions and therapeutics. World J. Biol. Psychiatry.

[3] Van den Bos M.A.J., Higashihara M., Geevasinga N., Menon P., Kiernan M.C., Vucic S. (2018). Imbalance of cortical facilitatory and inhibitory circuits underlies hyperexcitability in ALS. Neurology.

[4] Selten M., Van Bokhoven H., Kasri N.N. (2018). Inhibitory control of the excitatory/inhibitory balance in psychiatric disorders. F1000Research.

[5] Chen R., Yung D., Li J.-Y. (2003). Organization of ipsilateral excitatory and inhibitory pathways in the human motor cortex. J. Neurophysiol..

[6] Sanderson D.J., Good M.A., Seeburg P.H., Sprengel R., Rawlins J.N.P., Bannerman D.M. (2008). The role of the GluR-A (GluR1) AMPA receptor subunit in learning and memory. Prog. Brain Res..

[7] Shimshek D.R., Bus T., Schupp B., Jensen V., Marx V., Layer L.E., Köhr G., Sprengel R. (2017). Different forms of AMPA receptor mediated LTP and their correlation to the spatial working memory formation. Front. Mol. Neurosci..

[8] Mitsushima D., Ishihara K., Sano A., Kessels H.W., Takahashi T. (2011). Contextual learning requires synaptic AMPA receptor delivery in the hippocampus. Proc. Natl. Acad. Sci. USA.

[9] Qneibi M., Bdir S., Bdair M., Aldwaik S.A., Sandouka D., Heeh M., Idais T.I. (2024). AMPA receptor neurotransmission and therapeutic applications: A comprehensive review of their multifaceted modulation. Eur. J. Med. Chem..

[10] Niescier R.F., Lin Y.-C. (2021). The potential role of AMPA receptor trafficking in Autism and other neurodevelopmental conditions. Neuroscience.

[11] Babaei P. (2021). NMDA and AMPA receptors dysregulation in Alzheimer’s disease. Eur. J. Pharmacol..

[12] Egbenya D.L., Hussain S., Lai Y.-C., Xia J., Anderson A.E., Davanger S. (2018). Changes in synaptic AMPA receptor concentration and composition in chronic temporal lobe epilepsy. Mol. Cell. Neurosci..

[13] Kerr M.P. (2012). The impact of epilepsy on patients’ lives. Acta Neurol. Scand..

[14] Ostendorf A.P., Gedela S. (2017). Effect of Epilepsy on Families, Communities, and Society.

[15] Vazquez B., Devinsky O. (2003). Epilepsy and anxiety. Epilepsy Behav..

[16] Traynelis S.F., Wollmuth L.P., McBain C.J., Menniti F.S., Vance K.M., Ogden K.K., Hansen K.B., Yuan H., Myers S.J., Dingledine R. (2010). Glutamate receptor ion channels: Structure, regulation, and function. Pharmacol. Rev..

[17] Mayer M.L., Armstrong N. (2004). Structure and function of glutamate receptor ion channels. Annu. Rev. Physiol..

[18] Niciu M.J., Kelmendi B., Sanacora G. (2012). Overview of glutamatergic neurotransmission in the nervous system. Pharmacol. Biochem. Behav..

[19] Choi D.W. (1992). Excitotoxic cell death. J. Neurobiol..

[20] Zappala M., Grasso S., Micale N., Polimeni S., De Micheli C. (2001). Synthesis and structure-activity relationships of 2,3-benzodiazepines as AMPA receptor antagonists. Mini Rev. Med. Chem..

[21] Qneibi M., Jumaa H., Bdir S., Al-Maharik N. (2023). Electrophysiological Assessment of newly synthesized 2,3-Benzodiazepine derivatives for inhibiting the AMPA receptor Channel. Molecules.

[22] Qneibi M.S., Micale N., Grasso S., Niu L. (2012). Mechanism of inhibition of GluA2 AMPA receptor channel opening by 2,3-benzodiazepine derivatives: Functional consequences of replacing a 7,8-methylenedioxy with a 7,8-ethylenedioxy moiety. Biochemistry.

[23] Patel N.C. (2015). Perampanel (Fycompa): AMPA receptor antagonist for the treatment of seizure. Innov. Drug Synth..

[24] Steinhoff B.J. (2015). The AMPA receptor antagonist perampanel in the adjunctive treatment of partial-onset seizures: Clinical trial evidence and experience. Ther. Adv. Neurol. Disord..

[25] Guseynov A.D., Pisarev S.A., Shulga D.A., Palyulin V.A., Fedorov M.V., Karlov D.S. (2019). Computational characterization of the glutamate receptor antagonist perampanel and its close analogs: Density functional exploration of conformational space and molecular docking study. J. Mol. Model..

[26] Hawash M., Jaradat N., Abualhasan M., Şüküroğlu M.K., Qaoud M.T., Kahraman D.C., Daraghmeh H., Maslamani L., Sawafta M., Ratrout A. (2023). Design, synthesis, molecular docking studies and biological evaluation of thiazole carboxamide derivatives as COX inhibitors. BMC Chem..

[27] Bryson H.M., Fulton B., Benfield P. (1996). Riluzole: A review of its pharmacodynamic and pharmacokinetic properties and therapeutic potential in amyotrophic lateral sclerosis. Drugs.

[28] Hawash M. (2023). Thiazole Derivatives as Modulators of GluA2 AMPA Receptors: Potent Allosteric Effects and Neuroprotective Potential. Biomolecules.

[29] Rameau G.A., Tukey D.S., Garcin-Hosfield E.D., Titcombe R.F., Misra C., Khatri L., Getzoff E.D., Ziff E.B. (2007). Biphasic coupling of neuronal nitric oxide synthase phosphorylation to the NMDA receptor regulates AMPA receptor trafficking and neuronal cell death. J. Neurosci..

[30] Singh S., Loke Y.K., Furberg C.D. (2007). Thiazolidinediones and heart failure: A teleo-analysis. Diabetes Care.

[31] Loke Y.K., Singh S., Furberg C.D. (2009). Long-term use of thiazolidinediones and fractures in type 2 diabetes: A meta-analysis. CMAJ.

[32] Wallach J.D., Wang K., Zhang A.D., Cheng D., Nardini H.K.G., Lin H., Bracken M.B., Desai M., Krumholz H.M., Ross J.S. (2020). Updating insights into Rosiglitazone and cardiovascular risk through shared data: Individual patient and summary level meta-analyses. BMJ.

[33] Nissen S.E. (2010). The Rise and Fall of Rosiglitazone.

[34] Zarate C.A., Manji H.K. (2008). The Role of AMPA receptor modulation in the treatment of neuropsychiatric diseases. Exp. Neurol..

[35] Fontana A.C., Poli A.N., Gour J., Srikanth Y.V., Anastasi N., Ashok D., Khatiwada A., Reeb K.L., Cheng M.H., Bahar I. (2024). Synthesis and Structure—Activity Relationships for Glutamate Transporter Allosteric Modulators. J. Med. Chem..

[36] Qneibi M., Hawash M., Gümüş M., Çapan İ., Sert Y., Bdir S., Koca İ., Bdair M. (2023). Deciphering the Biophysical Properties of Ion Channel Gating Pores by Coumarin–Benzodiazepine Hybrid Derivatives: Selective AMPA Receptor Antagonists. Mol. Neurobiol..

[37] Charsouei S., Jabalameli M.R., Karimi-Moghadam A. (2020). Molecular insights into the role of AMPA receptors in the synaptic plasticity, pathogenesis and treatment of epilepsy: Therapeutic potentials of perampanel and antisense oligonucleotide (ASO) technology. Acta Neurol. Belg..

[38] Qneibi M., Jaradat N., Hawash M., Olgac A., Emwas N. (2020). Ortho versus Meta Chlorophenyl-2,3-Benzodiazepine Analogues: Synthesis, Molecular Modeling, and Biological Activity as AMPAR Antagonists. ACS Omega.

[39] Deng X.-Q., Song M.-X., Gong G.-H., Wang S.-B., Quan Z.-S. (2014). Synthesis and anticonvulsant evaluation of some new 6-(substituted-phenyl) thiazolo [3,2-b][1,2,4] triazole derivatives in mice. Iran. J. Pharm. Res. IJPR.

[40] Xi N., Bo Y., Doherty E.M., Fotsch C., Gavva N.R., Han N., Hungate R.W., Klionsky L., Liu Q., Tamir R. (2005). Synthesis and evaluation of thiazole carboxamides as vanilloid receptor 1 (TRPV1) antagonists. Bioorg. Med. Chem. Lett..

[41] Malik S., Bahare R.S., Khan S.A. (2013). Design, synthesis and anticonvulsant evaluation of N-(benzo [d] thiazol-2-ylcarbamoyl)-2-methyl-4-oxoquinazoline-3(4H)-carbothioamide derivatives: A hybrid pharmacophore approach. Eur. J. Med. Chem..

[42] Qneibi M., Hamed O., Jaradat N., Hawash M., Al-Kerm R., Al-Kerm R., Sobuh S., Tarazi S. (2021). The AMPA receptor biophysical gating properties and binding site: Focus on novel curcumin-based diazepines as non-competitive antagonists. Bioorg. Chem..

