# Supplementary material for: Assessing the Effects of Thiazole-Carboxamide Derivatives on the Biophysical Properties of AMPA Receptor Complexes as a Potential Neuroprotective Agent
Source: Molecules. 2024 Jul 8;29(13):3232. doi: 10.3390/molecules29133232 (PMC11243149; doi:10.3390/molecules29133232)
Supplement: Supplementary file 1 [file molecules-29-03232-s001.zip › molecules-3067848-supplementary.pdf]

Supplementary material

Assessing the Effects of Thiazole-Carboxamide Derivatives on the Biophysical Properties of AMPA Receptor Complexes as a Potential Neuroprotective Agent

Mohammad Qneibi [1,*], Mohammed Hawash [2], Sosana Bdir [1], Mohammad Bdair [1] and Samia Ammar Aldwaik [1]

1 Department of Biomedical Sciences, Faculty of Medicine and Health Sciences, An-Najah National University, Nablus, P400, Palestine; s12027767@stu.najah.edu (S.B.); mohammad.bdair02@gmail.com (M.B.); samiaammardweik@gmail.com (S.A.A.)

2 Department of Pharmacy, Faculty of Medicine and Health Sciences, An-Najah National University, Na-blus, P400, Palestine; mohawash@najah.edu

* Correspondence: mqneibi@najah.edu; Tel.: +972-545-975-016

Table of Contents

Tables S1-S5. Whole-Cell Recordings……………………………………………..…………..3-8

Table S6 IC_{50} values……………………….………………………...…………….………..9

Detailed Chemistry and Characterization of Thiazole-Carboxamide Derivatives (TC-1 to TC-5)……………………………....…………….....……………………………………10-11

The data shown is the mean ± SEM, with n = 10 being the number of patch cells in the whole-cell arrangement. A one-way ANOVA test was used to determine data significance, with thresholds of * $p < 0.05$, ** $p < 0.01$, and ns (not significant).

Table S1. Whole-Cell Recordings.

Receptor Name/Compounds abbreviation	GluA1 (Glutamate Alone)	TC-1	Applying Glutamate Alone After TC-1	n	A/A_I
Amplitude (pA)	867±84	177±20***	848±82	10	4.91±0.1
t deact (ms)	2.1±0.1	5.3±0.4***	N/R	10	N/R
t des (ms)	2.5±0.1	1.3±0.1***	N/R	10	N/R
**Receptor Name/Compounds abbreviation**	**GluA1/2 (Glutamate Alone)**	**TC-1**	**Applying Glutamate Alone After TC-1**	**n**	**A/A_I**
Amplitude (pA)	626±60	126±12***	604±62	10	4.98±0.06
t deact (ms)	2.5±0.3	5.8±0.4***	N/R	10	N/R
t des (ms)	5.2±0.5	3.9±0.4***	N/R	10	N/R
**Receptor Name/Compounds abbreviation**	**GluA2 (Glutamate Alone)**	**TC-1**	**Applying Glutamate Alone After TC-1**	**n**	**A/A_I**
Amplitude (pA)	1171±94	233±20***	1137±97	10	5.03±0.04
t deact (ms)	2.3±0.1	5.9±0.2***	N/R	10	N/R
t des (ms)	2.6±0.1	1.1±0.1***	N/R	10	N/R

Receptor Name/Compounds abbreviation	GluA2/3 (Glutamate Alone)	TC-1	Applying Glutamate Alone After TC-1	n	A/A_I
**Amplitude (pA)**	506±56	102±13***	491±61	10	4.95±0.19
**t deact (ms)**	2.6±0.3	5.6±0.4***	N/R	10	N/R
**t des (ms)**	2.7±0.2	1.2±0.3***	N/R	10	N/R

Table S2. Whole-Cell Recordings.

Receptor Name/Compounds abbreviation	GluA1 (Glutamate Alone)	TC-2	Applying Glutamate Alone After TC-2	n	A/A_I
**Amplitude (pA)**	860±91	172±18***	841±88	10	5.28±0.12
**t deact (ms)**	N/R	5.6±0.3***	N/R	10	N/R
**t des (ms)**	N/R	1.1±0.1***	N/R	10	N/R
**Receptor Name/Compounds abbreviation**	**GluA1/2 (Glutamate Alone)**	**TC-2**	**Applying Glutamate Alone After TC-2**	**n**	**A/A_I**
**Amplitude (pA)**	618±65	115±12***	597±66	10	5.38±0.08
**t deact (ms)**	N/R	6.0±0.5***	N/R	10	N/R
**t des (ms)**	N/R	3.7±0.3***	N/R	10	N/R
**Receptor Name/Compounds abbreviation**	**GluA2 (Glutamate Alone)**	**TC-2**	**Applying Glutamate Alone After TC-2**	**n**	**A/A_I**

**Amplitude (pA)**	1164±102	212±20***	1131±105	10	5.49±0.05
**t deact (ms)**	N/R	6.3±0.2***	N/R	10	N/R
**t des (ms)**	N/R	0.9±0.1***	N/R	10	N/R
**Receptor Name/Compounds abbreviation**	**GluA2/3 (Glutamate Alone)**	**TC-2**	**Applying Glutamate Alone After TC-2**	**n**	**A/A_I**
**Amplitude (pA)**	498±63	94±14***	484±68	10	5.33±0.23
**t deact (ms)**	N/R	5.9±0.4***	N/R	10	N/R
**t des (ms)**	N/R	1.0±0.2***	N/R	10	N/R

Table S3. Whole-Cell Recordings.

Receptor Name/Compounds abbreviation	GluA1 (Glutamate Alone)	TC-3	Applying Glutamate Alone After TC-3	n	A/A_I
**Amplitude (pA)**	849±103	287±38**	799±95	10	2.97±0.03
**t deact (ms)**	N/R	3.0±0.2*	N/R	10	N/R
**t des (ms)**	N/R	2.0±0.1ns	N/R	10	N/R
**Receptor Name/Compounds abbreviation**	**GluA1/2 (Glutamate Alone)**	**TC-3**	**Applying Glutamate Alone After TC-3**	**n**	**A/A_I**
**Amplitude (pA)**	608±74	197±24**	565±72	10	3.09±0.02
**t deact (ms)**	N/R	3.3±0.3*	N/R	10	N/R
**t des**	N/R	4.2±0.3*	N/R	10	N/R

**(ms)**					
**Receptor Name/Compounds abbreviation**	**GluA2 (Glutamate Alone)**	**TC-3**	**Applying Glutamate Alone After TC-3**	**n**	**A/A_I**
**Amplitude (pA)**	1153±114	363±37**	1078±112	10	3.17±0.02
**t deact (ms)**	N/R	3.5±0.3*	N/R	10	N/R
**t des (ms)**	N/R	1.7±0.1*	N/R	10	N/R
**Receptor Name/Compounds abbreviation**	**GluA2/3 (Glutamate Alone)**	**TC-3**	**Applying Glutamate Alone After TC-3**	**n**	**A/A_I**
**Amplitude (pA)**	488±73	159±25**	456±76	10	3.06±0.07
**t deact (ms)**	N/R	3.4±0.4**	N/R	10	N/R
**t des (ms)**	N/R	1.8±0.2**	N/R	10	N/R

Table S4. Whole-Cell Recordings.

**Receptor Name/Compounds abbreviation**	**GluA1 (Glutamate Alone)**	**TC-4**	**Applying Glutamate Alone After TC-4**	**n**	**A/A_I**
**Amplitude (pA)**	835±119	331±50*	816±114	10	2.53±0.02
**t deact (ms)**	N/R	2.9±0.1*	N/R	10	N/R
**t des (ms)**	N/R	2.1±0.1^{ns}	N/R	10	N/R
**Receptor Name/Compounds abbreviation**	**GluA1/2 (Glutamate Alone)**	**TC-4**	**Applying Glutamate Alone After TC-4**	**n**	**A/A_I**

**Amplitude (pA)**	631±72	221±26*	609±77	10	2.86±0.02
**t deact (ms)**	N/R	3.2±0.4*	N/R	10	N/R
**t des (ms)**	N/R	4.4±0.3*	N/R	10	N/R
**Receptor Name/Compounds abbreviation**	**GluA2 (Glutamate Alone)**	**TC-4**	**Applying Glutamate Alone After TC-4**	**n**	**A/A_I**
**Amplitude (pA)**	1143±99	393±43*	1110±101	10	2.91±0.07
**t deact (ms)**	N/R	3.4±0.2*	N/R	10	N/R
**t des (ms)**	N/R	1.8±0.1*	N/R	10	N/R
**Receptor Name/Compounds abbreviation**	**GluA2/3 (Glutamate Alone)**	**TC-4**	**Applying Glutamate Alone After TC-4**	**n**	**A/A_I**
**Amplitude (pA)**	473±90	183±36*	460±95	10	2.59±0.05
**t deact (ms)**	N/R	3.3±0.3*	N/R	10	N/R
**t des (ms)**	N/R	1.9±0.2*	N/R	10	N/R

Table S5. Whole-Cell Recordings.

Receptor Name/Compounds abbreviation	GluA1 (Glutamate Alone)	TC-5	Applying Glutamate Alone After TC-5	n	A/A_I
**Amplitude (pA)**	823±136	177±33**	823±136	10	4.67±0.10
**t deact (ms)**	N/R	5.5±0.4**	N/R	10	N/R
**t des**	N/R	1.4±0.1**	N/R	10	N/R

(ms)					
**Receptor Name/Compounds abbreviation**	**GluA1/2 (Glutamate Alone)**	**TC-5**	**Applying Glutamate Alone After TC-5**	**n**	**A/A$_I$**
**Amplitude (pA)**	590±92	123±20**	553±89	10	4.80±0.07
**t deact (ms)**	N/R	5.6±0.4**	N/R	10	N/R
**t des (ms)**	N/R	3.8±0.3**	N/R	10	N/R
**Receptor Name/Compounds abbreviation**	**GluA2 (Glutamate Alone)**	**TC-5**	**Applying Glutamate Alone After TC-5**	**n**	**A/A$_I$**
**Amplitude (pA)**	1135±135	250±35**	1071±134	10	4.90±0.04
**t deact (ms)**	N/R	5.8±0.3**	N/R	10	N/R
**t des (ms)**	N/R	1.2±0.1**	N/R	10	N/R
**Receptor Name/Compounds abbreviation**	**GluA2/3 (Glutamate Alone)**	**TC-5**	**Applying Glutamate Alone After TC-5**	**n**	**A/A$_I$**
**Amplitude (pA)**	470±94	99±22**	443±96	10	4.79±0.20
**t deact (ms)**	N/R	5.7±0.4**	N/R	10	N/R
**t des (ms)**	N/R	1.3±0.2**	N/R	10	N/R

Table S6. IC_{50} values.

**Receptor/Subunit**	**TC Derivatives**	TC-1	TC-2	TC-3	TC-4	TC-5
**GluA1**	**IC_{50}**	3.42	3.20	5.73	5.88	3.51
	**Log IC_{50}**	0.53	0.51	0.76	0.77	0.55
	**R square**	0.97	0.97	0.99	0.94	0.96

Receptor/Subunit	**TC Derivatives**	TC-1	TC-2	TC-3	TC-4	TC-5
**GluA1/2**	**IC_{50}**	3.36	3.10	5.64	5.78	3.49
	**Log IC_{50}**	0.53	0.49	0.75	0.76	0.54
	**R square**	0.97	0.96	0.97	0.97	0.99

Receptor/Subunit	**TC Derivatives**	TC-1	TC-2	TC-3	TC-4	TC-5
**GluA2**	**IC_{50}**	3.30	3.02	5.50	5.60	3.35
	**Log IC_{50}**	0.52	0.48	0.74	0.75	0.52
	**R square**	0.97	0.97	0.94	0.94	0.96

Receptor/Subunit	**TC Derivatives**	TC-1	TC-2	TC-3	TC-4	TC-5
**GluA2/3**	**IC_{50}**	3.38	3.04	5.53	5.64	3.42
	**Log IC_{50}**	0.53	0.48	0.74	0.75	0.53
	**R square**	0.97	0.96	0.99	0.99	0.98

Detailed Chemistry and Characterization of Thiazole-Carboxamide Derivatives (TC-1 to TC-5)

TC-1: 2-(4-Methoxyphenyl)-N-phenylthiazole-4-carboxamide

- **Purification**: Silica gel column chromatography using n-hexane: ethyl acetate solvent system (4:1).
- **Solid Product**: M.P. 138–140 °C, Yield: 74.5%.
- **IR (FTIR/FTNIR-ATR)**: 1675.08 cm^{-1} (amide carbonyl, C=O).
- **HRMS (m/z)**: $[M+H]^+$ calcd for $C_{17}H_{14}N_2O_2S$ 311.0650, found 311.0658.
- **^1H NMR (DMSO-d6)**: δ: 10.19 (1H, s, NH), 8.39 (1H, s, Ar–H), 8.10 (2H, d, J=8.5 Hz, Ar–H), 7.87 (2H, d, J=8 Hz, Ar–H), 7.39 (2H, t, J=8 Hz, Ar–H), 7.15–7.10 (3H, m, Ar–H), 3.86 (3H, s, $–OCH_3$).
- **^13C NMR (DMSO-d6)**: δ ppm: 167.73, 161.80, 159.52, 150.68, 138.83, 129.11, 128.82, 125.65, 124.68, 124.41, 120.96, 115.01, 55.93.

TC-2: N-(2,5-dimethoxyphenyl)-2-(4-methoxyphenyl)thiazole-4-carboxamide

- **Purification**: Silica gel column chromatography using n-hexane: ethyl acetate solvent system (4:1).
- **Solid Product**: M.P. 139.5–141.5 °C, Yield: 70.1%.
- **IR (FTIR/FTNIR-ATR)**: 1682.68 cm^{-1} (amide carbonyl, C=O).
- **HRMS (m/z)**: $[M+H]^+$ calcd for $C_{19}H_{18}N_2O_4S$ 371.1066, found 371.0918.
- **^1H NMR (DMSO-d6)**: δ: 9.87 (1H, s, NH), 8.42 (1H, s, Ar–H), 8.04 (1H, s, Ar–H), 7.97 (2H, d, J=7 Hz, Ar–H), 7.12 (2H, d, J=8.5 Hz, Ar–H), 7.05 (1H, d, J=9 Hz, Ar–H), 6.69 (1H, d, J=8.5 Hz, Ar–H), 3.92, 3.85, 3.74 (9H, s, $-OCH_3$).
- **^13C NMR (DMSO-d6)**: δ ppm: 168.14, 161.92, 158.53, 153.63, 150.14, 143.00, 128.51, 128.03, 125.25, 124.89, 115.26, 112.11, 108.38, 106.57, 57.06, 55.93, 55.83.

TC-3: N-(3,4-dimethoxyphenyl)-2-(4-methoxyphenyl)thiazole-4-carboxamide

- **Purification**: Silica gel column chromatography using n-hexane: ethyl acetate solvent system (4:1).
- **Solid Product**: M.P. 123.5–124.5 °C, Yield: 92.4%.
- **IR (FTIR/FTNIR-ATR)**: 1645.76 cm^{-1} (amide carbonyl, C=O).
- **HRMS (m/z)**: $[M+H]^+$ calcd for $C_{19}H_{18}N_2O_4S$ 371.1066, found 371.0882.
- **^1H NMR (DMSO-d6)**: δ: 10.06 (1H, s, NH), 8.34 (1H, s, Ar–H), 8.10 (2H, d, J=8.5 Hz, Ar–H), 7.54 (1H, s, Ar–H), 7.47 (1H, d, J=8.5 Hz, Ar–H), 7.11 (2H, d, J=8.5 Hz, Ar–H), 6.96 (1H, d, J=9 Hz, Ar–H), 3.86 (3H, s, $-OCH_3$), 3.79 (3H, s, $-OCH_3$), 3.76 (3H, s, $-OCH_3$).
- **^13C NMR (DMSO-d6)**: δ ppm: 167.69, 161.79, 159.19, 150.83, 148.96, 145.82, 132.34, 128.81, 125.67, 124.28, 115.01, 112.84, 112.31, 106.06, 56.17, 55.93.

TC-4: N-(4-(tert-butyl)phenyl)-2-(4-methoxyphenyl)thiazole-4-carboxamide

- **Purification**: Silica gel column chromatography using n-hexane: ethyl acetate solvent system (3:2).
- **Solid Product**: M.P. 135–137 °C, Yield: 75.6%.
- **IR (FTIR/FTNIR-ATR)**: 1681.58 cm^{-1} (amide carbonyl, C=O).
- **HRMS (m/z)**: $[M+H]^+$ calcd for $C_{21}H_{22}N_2O_2S$ 367.1480, found 367.1301.
- **^1H NMR (DMSO-d6)**: δ: 10.12 (1H, s, NH), 8.37 (1H, s, Ar–H), 8.09 (2H, d, J=8.5 Hz, Ar–H), 7.77 (2H, d, J=8.5 Hz, Ar–H), 7.40 (2H, d, J=8.5 Hz, Ar–H), 7.11 (2H, d, J=8.5 Hz, Ar–H), 3.86 (3H, s, $–OCH_3$), 1.30 (9H, s, t-butyl).
- **^13C NMR (DMSO-d6)**: δ ppm: 167.70, 161.79, 159.38, 150.79, 146.76, 134.24, 128.82, 125.72, 125.67, 124.51, 120.72, 115.01, 55.93, 34.56, 31.67.

TC-5: N-(3,5-dimethoxyphenyl)-2-(4-methoxyphenyl)thiazole-4-carboxamide

- **Purification**: Silica gel column chromatography using n-hexane: ethyl acetate solvent system (3.5:1.5).
- **Solid Product**: M.P. 156–158 °C, Yield: 66.7%.
- **IR (FTIR/FTNIR-ATR)**: 1664.89 cm^{-1} (amide carbonyl, C=O).
- **HRMS (m/z)**: $[M+H]^+$ calcd for $C_{19}H_{18}N_2O_4S$ 371.1066, found 371.1044.
- **^1H NMR (DMSO-d6)**: δ: 10.10 (1H, s, NH), 8.38 (1H, s, Ar–H), 8.10 (2H, d, J=8.5 Hz, Ar–H), 7.19 (2H, s, Ar–H), 7.11 (2H, d, J=8.5 Hz, Ar–H), 6.30 (1H, s, Ar–H), 3.86 (3H, s, $-OCH_3$), 3.76 (6H, s, $-OCH_3$).
- **^13C NMR (DMSO-d6)**: δ ppm: 167.77, 161.82, 160.89, 159.52, 150.57, 140.51, 128.85, 125.62, 124.81, 115.00, 99.08, 96.50, 55.93, 55.65.
